# Leukemia relapse after transplantation – a consensus on monitoring, prevention, and treatment in China

**DOI:** 10.1186/s12916-019-1273-1

**Published:** 2019-02-12

**Authors:** Huichun Zhan

**Affiliations:** 1 Division of Hematology and Oncology, Department of Medicine, Stony Brook School of Medicine, Stony Brook University, Stony Brook, NY 11794 USA; 2 Northport VA Medical Center, Northport, New York, USA

**Keywords:** Stem cell transplantation, Leukemia, Relapse

## Background

Allogeneic hematopoietic stem cell transplantation (allo-HSCT) is the only curative treatment for many patients with hematologic malignancies. However, relapse after allo-HSCT remains a major challenge, and patients with relapsed disease usually have a dismal prognosis. Recently, on behalf of the HSCT working group of the Chinese Society of Hematology and Chinese Medical Association, experts from major transplant centers in China came together and produced the Chinese consensus on the management of post-HSCT leukemia relapse, which was published in *Cancer Letters* [1]. In this consensus, Wang et al. focused on three critical components of leukemia relapse after allo-HSCT: monitoring of minimal residual disease, prevention, and treatment of relapse. This consensus not only offers an overview of the substantial progress made by Chinese physicians and scientists in leukemia treatment, but also provided an opportunity to compare the different strategies adopted in different parts of the world.

## A consensus unique to China

Compared to the recommendations made by the National Cancer Institute of the United States [2, 3] and the European Society for Blood and Marrow Transplantation [4], the Chinese consensus is unique in the following areas:

1. Operational recommendations for minimal residual disease cut-off levels and the detection time-point for Chinese patient populations, such as the combination of quantitative WT1 expression and multiparameter flow cytometry assay for leukemia-associated aberrant immunophenotypes
2. Novel immunologic interventions developed by Chinese groups; for example, the modified donor lymphocyte infusion (DLI) protocol introduced by Huang in 2003 [5], which includes infusion of G-CSF-mobilized peripheral blood progenitor cells followed by short-term immunosuppressive agents to enhance the graft-versus-leukemia effects while decrease DLI-related toxicities
3. An individualized, risk-adapted strategy for prophylaxis, pre-emptive intervention, and treatment of leukemia relapse post-HSCT.

These consensus recommendations will help standardize the transplant practices in China, where 6601 allo-HSCT procedures were performed in 2017 [6] (compared with 6,189 allo-HSCT in Europe and 8,351 in the USA in 2015 [7]).

The modified DLI protocol and the large number of patients treated with this protocol (now part of a consensus recommendation) also provide a unique opportunity to investigate the mechanisms of disease relapse after allo-HSCT, which is a major cause of morbidity and mortality in patients with leukemia. For example, how do different donor types, conditioning regimens, post-transplant therapies, or immunomodulatory interventions affect the malignant stem cells and their microenvironment in different disorders? What are the mechanisms for normal/malignant cell competition, especially during the early stage of disease relapse?

It is anticipated that the implementation of such consensus recommendations will not only improve the care of many patients with leukemia and other hematologic malignancies in China, but also lead to a better understanding of these disorders through bench to bedside to bench research, which is essential for the development of novel, more effective therapeutic strategies for our patients. However, a cautious approach towards implementation is warranted. As discussed by Wang et al. [1], most of the recommendations were based on level 2A evidence, which is considered low-level. Therefore, within the consensus statement, the expert panel had made strong recommendations to enroll patients onto well-designed clinical trials as “the first-choice whenever possible for relapse post allo-HSCT”, highlighting a major challenge in clinical research in China.

## Conclusions

The diagnosis and management of Chinese cancer patients has mostly been adopted from foreign guidelines. Recent studies have illustrated that the different genetic background in the Asian population has significant consequences in cancer diagnosis, treatment, and prognosis [8]. For better patient care in China, there is therefore an urgent need to develop clinical guidelines using specific Chinese patient populations. It is reassuring to know that the Chinese consensuses on various benign and malignant hematologic disorders (including this consensus on the management of post-HSCT leukemia relapse) have been developed by our Chinese colleagues [9, 10]. Implementation of these consensus recommendations into clinical practice, and continued updates of these guidelines, is of equal or greater importance in China. Our patients deserve nothing less.

## Abbreviations

Allo-HSCT: Allogeneic hematopoietic stem cell transplantation
DLI: Donor lymphocyte infusion
HSCT: Hematopoietic stem cell transplantation
